# BAS2 Is Required for Conidiation and Pathogenicity of *Colletotrichum gloeosporioides* from *Hevea brasiliensis*

**DOI:** 10.3390/ijms19071860

**Published:** 2018-06-25

**Authors:** Bang An, Wenfeng Wang, Yunfeng Guo, Qiannan Wang, Hongli Luo, Chaozu He

**Affiliations:** Hainan Key Laboratory for Sustainable Utilization of Tropical Bioresources, Institute of Tropical Agriculture and Forestry, Hainan University, Haikou 570228, China; wangwenfenghn@163.com (W.W.); GUOYUNFENG18689731093@outlook.com (Y.G.); wangqiannan@hainu.edu.cn (Q.W.); hlluo@hainu.edu.cn (H.L.)

**Keywords:** *Colletotrichum gloeosporioides*, biotrophy-associated secreted protein, conidiation, pathogenicity, extracellular proteomics

## Abstract

The hemibiotrophic fungi *Colletotrichum gloeosporioides* can cause anthracnose in rubber trees. By searching the genome of the fungal pathogen, the *BAS2* encoding a biotrophy-associated secreted protein was identified. In the present study, the knockout mutants of *BAS2* were constructed and the functions of *BAS2* were investigated. The in vitro assays showed that BAS2 was not necessary for vegetative growth but was important for normal asexual reproduction in *C. gloeosporioides*. Pathogenicity assays suggested that BAS2 was involved in the process of the pathogen penetrating into the host tissue. Subcellular localization analysis revealed that BAS2 showed secretional characteristics in the fungi, and BAS2 mainly function as a cytoplasmic protein after being secreted into the host cell. Extracellular proteomics analysis revealed that BAS2 was required for the secretion of a series of proteins, which were important for the pathogenicity of *C. gloeosporioides*. These data lead to a better understanding of the biotrophy-associated secreted protein in regulating the pathogenesis of *C. gloeosporioides*.

## 1. Introduction

Anthracnose caused by the fungal pathogen *Colletotrichum gloeosporioides* is one of the main plant diseases in the rubber tree (*Hevea brasiliensis*), which leads to decreased rubber yield and serious economic losses to rubber planting. A hemibiotrophic lifestyle is a distinctive aspect of *Colletotrichum* species. After penetrating into the host epidermis, most *Colletotrichum* species initially colonize living plant tissue and obtain nutrients from living host cells. After a period of time, *Colletotrichum* switch to necrotrophy, leading to massive cell death and tissue destruction in the host. To overcome the plant’s defense systems, plant pathogens employ effector proteins as molecular weapons to influence the physical processes of the host [1,2,3]. Genome and transcriptome analyses revealed that a series of effector proteins were induced before penetration and during biotrophy in *Colletotrichum* species [4]. It has been reported that the *CgDN3* gene [5] was implicated in the biotrophic phase and required for suppressing host resistance responses in *C. gloeosporioides*, causing Stylosanthes anthracnose. In *Colletotrichum higginsianum*, the extracellular LysM effector proteins play dual roles in appressorial function and suppression of chitin-triggered plant immunity [6]. The fungalysin metalloprotease is specifically expressed during the biotrophic stage of infection and is involved in the development of infected mycelium in *Colletotrichum graminicola* [7]. The effector, CtNUDIX, can disrupt plant cell plasma membrane integrity and cell death, leading to a switch of lifestyle from biotrophic mode to necrotrophy in *Colletotrichum truncatum* [8].

Most plant pathogenic fungi differentiate infection structures, named appressoria, to penetrate the cuticle and cell wall of their hosts; after successful penetration, other specialized structures, called primary hyphae, develop and differentiate into biotrophic invasive hyphae. Previous work suggested that appressoria, primary hyphae, and invasive hyphae were the main structures for effector protein secretion in *Magnaporthe oryzae* [9]. Among these effector proteins, four proteins, named MoBAS1–4, were confirmed to be fungal biotrophy-associated secreted proteins in *M. oryzae* [10]. Transcriptional analysis showed that MoBAS were all upregulated in invasive hyphae. Protein sequence analysis revealed that MoBAS2, MoBAS3, and MoBAS4 encode secreted proteins with conserved Cys residues. In the genome of *C. gloeosporioides* from *H. brasiliensis*, the gene *BAS2* encoding homologous protein to MoBAS2 was identified. In our previous work, deletion of part of the coding sequence of *BAS2* led to decreased pathogenicity of *C. gloeosporioides*, but the mechanism of BAS2 in regulating the virulence of the pathogen remains unclear; besides, whether BAS2 is involved in vegetative growth and conidiation is still unknown. Therefore, in the present study, the coding sequence of *BAS2* was completely deleted from the *C. gloeosporioides* genome, and the functions of BAS2 in regulating growth and pathogenicity and the possible mechanism were investigated.

## 2. Results

### 2.1. Generation of the ∆BAS2 Mutant

In our previous work, knocking out part of the BAS2 coding sequence led to decreased pathogenicity of *C. gloeosporioides* to *H. brasiliensis* leaves [11]. To fully understand the functions of BAS2, the nucleotide sequence of BAS2 was fully deleted from the genome of *C. gloeosporioides* using the homologous recombination strategy (Supplementary Figure S3A). After the transformation, the Hygromycin-resistant colonies were analyzed with PCR to ensure correct integration into the gene locus. As shown in Supplementary Figure S3A, primer pairs with one primer outside of the flanking region and one inside the replacement fragments were used. The results show that the replacement fragments were correctly integrated into the BAS2 locus in the mutants. The knockout mutants were named ∆BAS2. Then the transformants were purified by single conidia isolation to exclude heterokaryons.

### 2.2. BAS2 Is Involved in Vegetative Growth and Conidiation

**Figure 1:**
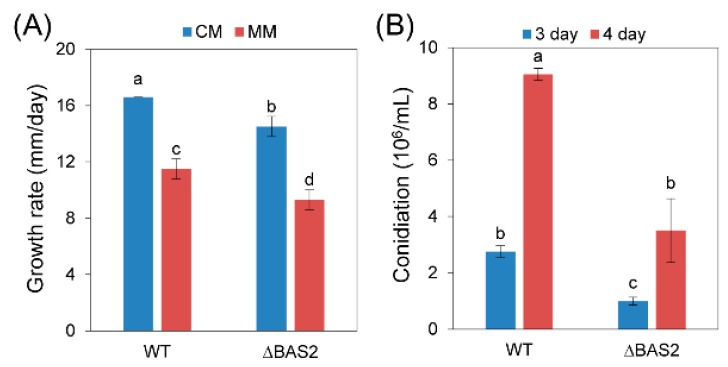
Growth rate and conidiation assays. (**A**) Growth rate of wild-type (WT) and ΔBAS2 cultured on complete medium (CM) and minimal medium (MM); (**B**) conidiation of WT and ΔBAS2. Bars represent standard deviation (SD). Columns with different letters indicate significant difference (*p* < 0.05).

As shown in Figure 1A, knocking out of BAS2 led to mild inhibition of vegetative growth in vitro. After being cultured in liquid complete medium for 3 and 4 days, the concentrations of conidia of the wild-type (WT) strain were up to 2.8 × 10^6^ and 9.1 × 10^6^ conidia/mL, while those of ∆BAS2 were only 1.0 × 10^6^ and 3.5 × 10^6^ conidia/mL (Figure 1B), suggesting significant impairment of conidia generation of the mutant.

### 2.3. BAS2 Are Required for Pathogenicity

**Figure 2:**
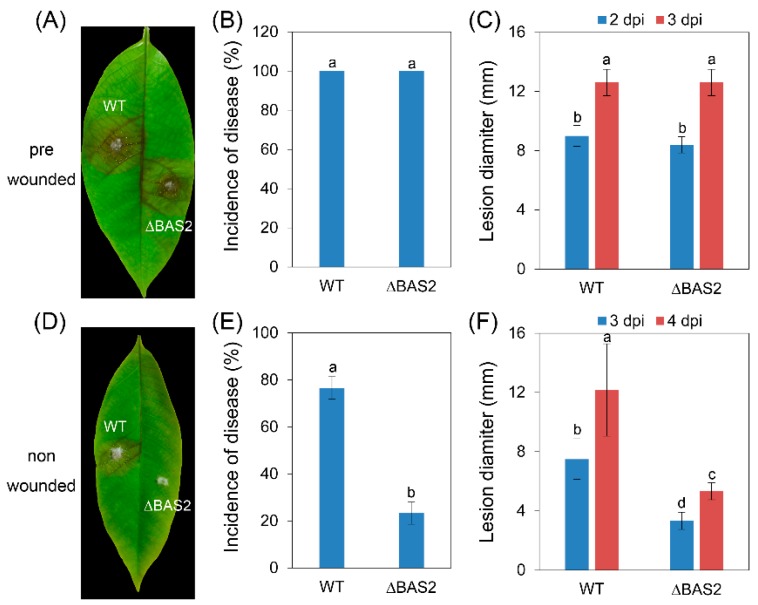
Pathogenicity assay on rubber-tree leaves. (**A**,**D**) Disease symptoms of rubber-tree leaves at 3 days post-inoculation (dpi). (**B**,**E**) Mean incidence of disease of rubber-tree leaves at 3 dpi. (**C**,**F**) Mean lesion diameters after at different dpi. (**A**–**C**) Rubber-tree leaves were prewounded before the inoculation. (**D**–**F**) Rubber-tree leaves were nonwounded before the inoculation. Bars represent standard deviation (SD). Columns with different letters indicate significant difference (*p* < 0.05).

To test whether pathogenicity and penetration ability were impaired in ∆BAS2, the pathogens were inoculated on leaves that were prewounded or nonwounded. The results show that when inoculated on prewounded leaves, both WT and ∆BAS2 showed 100% incidence of disease (Figure 2B), and the lesions caused by ∆BAS2 and by WT had nearly the same diameter (Figure 2C). When inoculated on nonwounded leaves, the incidence of disease of ∆BAS2 decreased to 23%, while that of WT was still up to 77% (Figure 2E); furthermore, the lesions caused by ∆BAS2 were about half the diameter of those caused by WT (Figure 2F).

### 2.4. Subcellular Localization of BAS2-GFP in C. gloeosporioides

**Figure 3:**
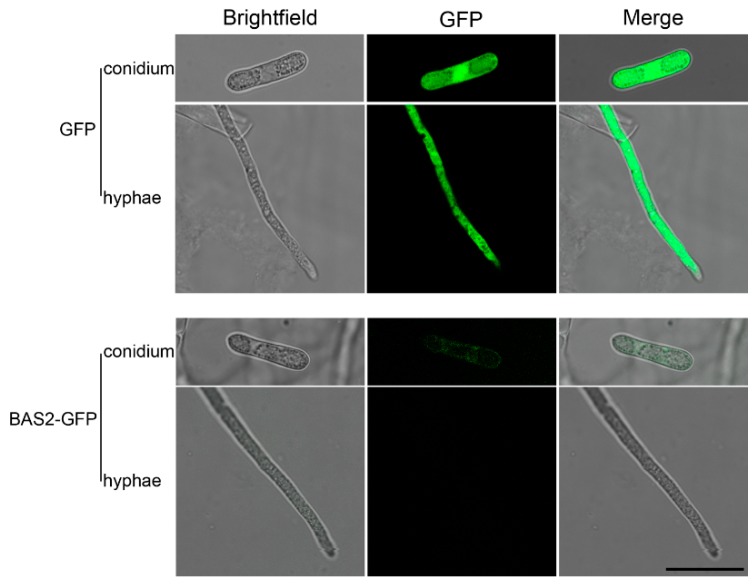
Subcellular localization of BAS2-green fluorescent protein (GFP) in conidia and hyphae of *C. gloeosporioides*. Scale bar = 20 μm.

In the present study, the nitrate reductase (*niaD*) gene locus was used to construct the BAS2-green fluorescent protein (GFP) fusion expressing transformants with the homologous recombination strategy (Supplementary Figure S4). The preliminary experiments showed that niaD is not required for vegetative growth or full virulence in *C. gloeosporioides* (Supplementary Figure S5), and similar phenotypes were found in the BcniaD deletion mutant of *Botrytis cinerea* [12]. Hence, the vector pCgNDHTN was suitable for the reporter gene constructs. The BAS2-GFP expressing transformant was constructed using pCgNDHTN. The transformants expressing only GFP showed strong fluorescence all through the cytoplasm in *C. gloeosporioides*. In contrast, the transformants expressing the BAS2-GFP fusion protein only showed weak fluorescence in conidia; furthermore, no fluorescence was observed in the hyphae (Figure 3), suggesting that BAS2 was secreted out of the cell.

### 2.5. Subcellular Localization of BAS2-GFP in Mesophyll Cell Protoplasts of H. brasiliensis

**Figure 4:**
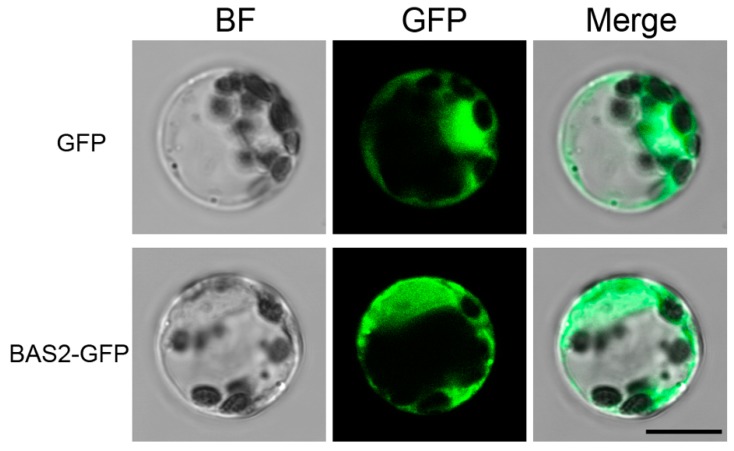
Subcellular localization of BAS2-GFP in mesophyll cell protoplasts of *H. brasiliensis*. Scale bar = 10 μm.

Transient expression of BAS2-GFP in mesophyll cell protoplasts of *H. brasiliensis* was conducted to detect the subcellular localization of BAS2-GFP in host cells. As shown in Figure 4, fluorescence of BAS2-GFP was observed all through the cytoplasm of the protoplasts of *H. brasiliensis*, showing the same pattern as the control that expressed empty GFP, suggesting that BAS2 was localized in cytoplasm in the host cell.

### 2.6. BAS2 Is Involved in Extracellular Protein Secretion

**Figure 5:**
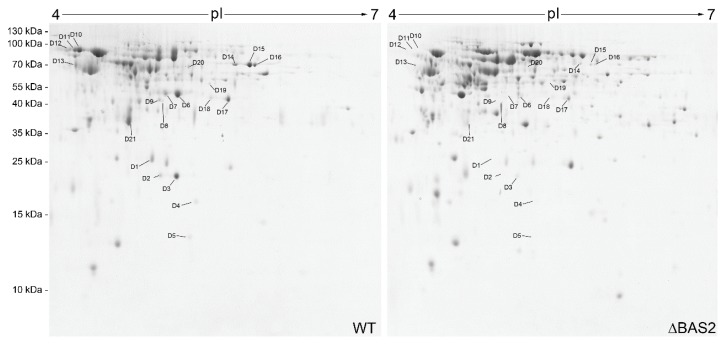
Two-dimensional patterns of extracellular proteomes of WT and ΔBAS2. Arrows indicate protein spots that were downregulated in abundance more than 1.5-fold between WT and ΔBAS2. Numbering of protein spots corresponds to numbering in Table 1.

To investigate whether BAS2 was involved in the protein secretion process, comparative extracellular proteomics analysis was conducted. Two-dimensional (2D) gel electrophoresis was conducted, and a total of 420 protein spots were detected on the Coomassie brilliant blue stained 2D gels, including samples from both WT and ∆BAS2. Quantitative image analysis revealed that 21 protein spots were downregulated (Figure 5) and 51 were upregulated (Supplementary Figure S6) in abundance in ∆BAS2. The 22 downregulated protein spots were excised and submitted to tandem mass spectrometry and identified by database searching with the Mascot search engine (Table 1). The identified proteins were classified into six functional categories according to their biological function: cell structure, redox, metabolism, secondary metabolism, cell wall degradation, and unknown.

## 3. Discussion

In recent years, an increasing number of effectors have been identified in fungal pathogens. Four Cys-rich secreted proteins related to biotrophy were identified and named biotrophy-associated secreted (BAS) proteins in *M. oryzae* [10]. By searching the genome, one gene-encoding protein homologous to MoBAS2 in *C. gloeosporioides* from *H. brasiliensis* was identified and named *BAS2*. Sequence alignment of the predicted BAS2 proteins of *C. gloeosporioides* from *H. brasiliensis*, *Colletotrichum gloeosporioides* Nara gc5, *C. higginsianum*, *C. graminicola*, *M. oryzae*, and *Podospora anserine* revealed that BAS2 proteins are well conserved in filamentous fungi (Supplementary Figures S1 and S2).

For functional analysis, knockout mutants of *BAS2* were constructed (Supplementary Figure S3). The growth and conidiation of ∆BAS2 were analyzed in vitro. The results show that only a very slight decrease in growth rate was observed in ∆BAS2 compared with WT, but conidia generation of ∆BAS2 was reduced about three times compared with WT; these results reveal that BAS2 is not necessary for vegetative growth but is important for normal asexual reproduction in *C. gloeosporioides*. Successful penetration into the host tissue is the first step of pathogenicity. To test whether BAS2 is involved in the penetration process, *C. gloeosporioides* strains were inoculated on leaves in two ways, with leaves prewounded or nonwounded. The results show that when inoculated on the wounds of leaves, both WT and ∆BAS2 caused 100% incidence of disease, but when inoculated on nonwounded leaves, the incidence of ∆BAS2-caused disease decreased sharply compared with WT, suggesting that BAS2 is important for the penetration process of pathogen to host. Besides, the diameters of lesions caused by ∆BAS2 were nearly the same as those caused by WT when inoculated on prewounded leaves, suggesting that BAS2 is not required for mycelial growth in plant tissue. When inoculated on nonwounded leaves, the diameters of lesions caused by ∆BAS2 were smaller than those caused by WT, but this might be due to decreased penetration ability of ∆BAS2. However, deletion of MoBAS in *M. oryzae* did not show a change in mycelial growth, conidiation, or pathogenicity [10].

Subcellular localization analysis showed that MoBAS2 localized to the biotrophic interfacial complex (BIC) of invasive hyphae in *M. oryzae* and had secretion characteristics [10,13]. In the present study, transformants overexpressing BAS2-GFP fusion protein were constructed. Microscopic analysis showed that little fluorescence was observed in the hyphae of the transformants, suggesting that BAS2 functions as a secreted protein in *C. gloeosporioides* from *H. brasiliensis*. After being secreted into the host cell, effector proteins could function as cytoplasmic proteins, bind to the plasma membrane proteins [14], and even transfer into the nuclei [15]. To investigate the subcellular localization of BAS2 in the host cells, transient expression of BAS2-GFP in the protoplasts of *H. brasiliensis* was conducted, and the results show that BAS2 was localized in cytoplasm in *H. brasiliensis* cells.

Effector proteins could not only directly bind to the targets in the host cell to disturb the host immune system, but also act as regulatory proteins to control the physical activities of pathogens themselves [16]. In the present study, BAS2 was involved in the penetration process of *C. gloeosporioides* from *H. brasiliensis*, suggesting its close relation to the protein secretion process. Therefore, the extracellular proteome of ∆BAS2 was analyzed. The results show that a total of 21 protein spots representing 12 proteins were significantly downregulated in the 2D gel images of ∆BAS2 (Figure 5). Interestingly, another 51 protein spots were upregulated in ∆BAS2 (Supplementary Figure S6). Since deletion of BAS2 led to decreased conidiation and pathogenicity, only the downregulated protein spots were investigated in subsequent analysis. Among the 12 proteins, 2 enzymes involved in cell wall degradation were downregulated in the mutant. *Colletotrichum* species use a combination of mechanical force and enzymatic degradation to penetrate into the host surfaces. Therefore, a reduced abundance of the two enzymes might be the cause of decreased disease incidence in ∆BAS2 (Figure 2E). Oxidative burst is one of the earliest events in the plant’s hypersensitive response to pathogen attack. In the previous work, we found that inoculation of *C. gloeosporioides* could cause a significant increase of hydrogen peroxide accumulation in *H. brasiliensis* leaves [17]. The proteome analysis here showed that *C. gloeosporioides* secreted abundant peroxidase to the extracellular spaces, suggesting that peroxidase plays an important role in regulating the redox level of the host tissue. In ∆BAS2, the abundance of extracellular peroxidase decreased dramatically, which could lead to decreased disease incidence and lesion development (Figure 2E,F). It is well known that plant pathogens could secret auxin to facilitate pathogenicity to host plants [18,19]. Here we found that indoleacetamide hydrolase (IaaH), one of the key enzymes for auxin synthesis in fungi, was secreted to the host cell by *C. gloeosporioides*. However, deletion of *BAS2* caused a dramatic decrease in the abundance of the enzyme, which might be another cause of the decreased pathogenicity in ∆BAS2. Melanin is a secondary metabolite in fungi, which plays important roles in conferring tolerance to environmental stresses, differentiating conidia and appressoria, and pathogenicity to the host [20,21]. In ∆BAS2, tyrosinase, one of the key enzymes in the biosynthesis of melanin, was downregulated significantly. Besides, the protein abundance of versicolorin b synthase, which is involved in aflatoxin biosynthesis, was also decreased in ∆BAS2. Actin, a component of intracellular cytoskeleton, was also identified from the extracellular proteome of *C. gloeosporioides*, suggesting that there might be autophagic fungal cell death during the infection process [22]. However, the actin protein was not observed in ∆BAS2, indicating decreased infection ability of the mutant. Moreover, five identified proteins involved in metabolism were also downregulated in ∆BAS2, suggesting that the pathogen could also interfere with the normal metabolism of the host cell. The previous studies revealed that *C. graminicola* gene expression is highly dynamic and is induced in successive waves during specific stages of the infection process [4].

Taken together, BAS2 is involved in the secretion of proteins closely related to pathogenicity and plays an important role in conidiation and pathogenicity of *C. gloeosporioides* from *H. brasiliensis*.

## 4. Materials and Methods

### 4.1. Fungal Strains and Culture Conditions

*C. gloeosporioides* strain was isolated from *H. brasiliensis* leaves with anthracnose and grown on potato dextrose agar (PDA) at 28 °C.

### 4.2. Construction of the Knockout Vector of BAS2

To construct the knockout vector of BAS2, plasmid pBluescript II KS (+) (pBS) was used as the backbone. First, the Hygromycin phosphotransferase gene expressing cassette (*HPH*) was amplified from the vector pKoV21 and ligated into pBSat of the XbaI/EcoRI sites to construct the vector pBS-*HPH*. Then the 5′ and 3′ flanking regions of the BAS2 gene were amplified from genomic DNA with the restriction sites added at both ends of flank, as shown in Supplementary Figure S3A. After that, the 2 flanking regions were ligated into the vector pBS-*HPH* at the corresponding sites to yield the knockout vector. The knockout vector was linearized with SacI (Thermo Fisher Scientific, Waltham, MA, USA) before transformation of the *C. gloeosporioides* strain. All the primers used are listed in Supplementary Table S1.

### 4.3. Transformation of C. gloeosporioides, PCR Diagnosis, and Single Conidia Purification

Protoplast formation and transformation of *C. gloeosporioides* were performed using the methods described by Wang et al. [17]. The Hygromycin-resistant strains were isolated and analyzed by PCR with the primer pairs shown in Supplementary Figure S3, which are diagnostic for homologous integration of the 5′ part and 3′ part. Then the correct transformants were purified by single conidia isolations.

### 4.4. Growth and Conidiation Assay

The *C. gloeosporioides* strains were inoculated on complete medium and minimal medium [23] and grown for 5 days. The diameters of the colonies were recorded and the growth rates were calculated. Conidia were harvested from *C. gloeosporioides* strains grown on PDA medium, washed with ddH_2_O, and inoculated into 50 mL liquid complete medium, making an initial concentration of 10^3^ conidia/mL. Then the strains were cultured at 28 °C at 150 rpm for the desired time, and the conidia number was calculated under a microscope.

### 4.5. Pathogenicity Assay

The *C. gloeosporioides* strains were grown on PDA medium for 2 days, and pieces of mycelia together with agar (about 1 mm^3^) were excised from the growing edge of the colonies. The *H. brasiliensis* leaves at the “light green” stage were detached from rubber tree Varity 73-3-97, and half of the leaves were wounded with sterilized needles. For the pathogenicity assay, mycelia were inoculated on the leaves that were prewounded or nonwounded. After that, the inoculated leaves were kept in a moist chamber at 28 °C under natural illumination for 4 days and the disease symptoms were scored. Each treatment contained 3 replicates of 10 leaves, and the entire experiment was repeated twice.

### 4.6. Generation of BAS2-GFP Fusion Expressing Mutant

To construct an efficient expression system for *C. gloeosporioides*, the vector pCgNDHTN was constructed according to Schumacher [12]. Briefly, the gene locus of *niaD* was used for targeted integration of reporter gene constructs together with promoter and terminator. The strategy for the vector construction is shown in Supplementary Figure S4A. First, the 5′ and 3′ flanking regions of the *niaD* were amplified and ligated into the vector pBS; second, the *HPH* cassette was amplified and ligated into the vector pBS; finally, the promoter of *ToxA* and the terminator of *nos* were amplified from the vector pCT74 [24] and ligated into the vector pBS to generate pCgNDHTN.

Green fluorescent protein (GFP) was used as the reporter gene to analyze the subcellular location of BAS2 in *C. gloeosporioides*. To construct the BAS2-GFP fusion vector, the open reading frame of BAS2 and the coding sequence of GFP with an N-terminal linker [12] were amplified and ligated into the PstI/SpeI and SpeI/XbaI sites of pCgNDHTN, respectively. The BAS2-GFP fusion vector was then named pCgNDHTN-BAS2-GFP (Supplementary Figure S4B), and the vector was linearized with SacI before transformation of *C. gloeosporioides*. The transformants were analyzed by PCR to ensure correct integration into the *niaD* gene locus. Then the correct mutants expressing BAS2-GFP were grown in the liquid complete medium, and the subcellular localization of BAS2-GFP fusion protein in conidia and hyphae of *C. gloeosporioides* was observed under a Leica TCS SP8 laser scanning confocal microscope, with argon laser excitation of 488 nm and an emission wavelength range of 505–525 nm. The intensity of the argon laser in laser configuration and intensity of laser line 488 in the acquire section were set to 20% and 15%, respectively. Pinhole was set to 1.8 Airy units. To compare the fluorescence intensity of GFP in samples, all optical sections were acquired under identical conditions.

### 4.7. Construction of BAS2-GFP Fusion Expressing Vector and Transient Expression in Mesophyll Cell Protoplasts of H. brasiliensis

The BAS2-GFP fusion expressing vector was constructed by ligating the open reading frame of BAS2 and the GFP sequence into the downstream of the cauliflower mosaic virus 35S promoter. The rubber-tree mesophyll cell protoplasts were prepared and transformed according to Zhang et al. [25]. The subcellular localization of BAS2-GFP fusion protein in mesophyll cell protoplasts of *H. brasiliensis* was observed under the confocal, with the protoplasts expressing GFP alone as the empty control.

### 4.8. Extracellular Proteomic Analysis

To induce the expression of extracellular proteins, modified Czapeck liquid medium in which glucose was replaced with apple pectin was used to culture *C. gloeosporioides*. *C. gloeosporioides* strains were grown for 7 days on PDA medium, then the conidia were collected, washed 2 times with ddH_2_O, and inoculated into 200 mL Czapeck liquid medium to make an initial concentration of 10^3^ conidia/mL. After being cultured for 3 days at 28 °C at 160 rpm, the supernatant was filtrated and centrifuged to remove impurities. The extracellular proteins were precipitated from the supernatant by adding sodium deoxycholate (Sigma-Aldrich) to the final concentration of 0.03% (*w*/*v*), followed by incubation on ice for 30 min; subsequently, trichloric acetic acid (100%) (Sigma-Aldrich, St. Louis, MO, USA) was added to the mixture to a final concentration of 10% (*v*/*v*), followed by incubation on ice for another 30 min. Then the precipitated proteins were collected by centrifugation at 16,000× *g* at 4 °C for 30 min and washed 3 times with cold acetone. To remove residual apple pectin, the air-dried precipitate was redissolved in extraction buffer (0.5 M Tris-HCl, pH 8.3, 2% (*v*/*v*) NP-40, 20 mM MgCl_2_, 2% (*v*/*v*) b-mercaptoethanol, and 1 mM Phenylmethanesulfonyl fluoride), and an equal volume of Tris-HCl (pH 7.8) buffered phenol was added into the mix to extract proteins. After centrifugation, proteins were precipitated from the final phenol phase with 5 vol of ice-cold saturated ammonium acetate in methanol overnight at −20 °C. Then the proteins were collected by centrifugation and washed twice with cold saturated ammonium acetate in methanol and acetone. The air-dried proteins were solubilized in the thiourea/urea lysis buffer, and 2D gel electrophoresis and MALDI-TOF/TOF mass spectrometer analyses were conducted according to Wang et al. [17].

## Figures and Tables

**Table 1:** Proteins identified by quadrupole-time-of-flight tandem mass spectrometry. The accession numbers of the proteins of *C. gloeosporioides* Nara gc5, which showed the most homology to the protein spots, are listed. NP, number of matched peptides; SC, amino acid sequence coverage for identified proteins; WT vs. ΔBAS2, average fold change of relative abundance of specific spot of WT versus ΔBAS2 from three biological repeats; **∞**, corresponding spot appeared in WT but not in ΔBAS2.

Spot	Protein Function	Accession Number	NP	SC (%)	WT vs. ∆BAS2	Biological Function
Cell structure						
D1	Actin	ELA34037.1	2	6	∞	Cytoskeleton
Redox						
D6	Peroxidase	ELA30823.1	14	37	38.56	Redox
D8	Peroxidase	ELA30823.1	11	30	∞	Redox
D9	Peroxidase	ELA30823.1	12	34	∞	Redox
Metabolism						
D7	Endonuclease/exonuclease/phosphatase family protein	ELA30823.1	13	35	∞	Metabolism
D11	Choline dehydrogenase	ELA24295.1	10	17	∞	Choline metabolism
D12	Choline dehydrogenase	ELA24295.1	9	16	∞	Choline metabolism
D13	Flavine adenine dinucleotide-dependent oxygenase	ELA25647.1	10	22	33.04	Metabolism
D15	Flavine adenine dinucleotide-binding dehydrogenase	ELA24979.1	3	5	13.20	Metabolism
D21	Enolase	ELA34631.1	15	30	14.04	Glycolytic pathways
Secondary metabolism						
D10	Indoleacetamide hydrolase (IaaH)	ELA36057.1	8	14	∞	IAA synthesis
D14	Versicolorin b synthase	ELA30672.1	13	19	26.33	Aflatoxin synthesis
D16	Versicolorin b synthase	ELA30672.1	16	25	45.11	Aflatoxin synthesis
D19	Tyrosinase central domain containing protein	ELA33276.1	4	8	∞	Melanin synthesis
**Cell** wall degradation						
D17	Endo-β-1,6-glucanase	ELA27535.1	11	21	1.70	Degradation of chitin
D18	Endo-β-1,6-glucanase	ELA27535.1	8	19	∞	Degradation of chitin
D20	Exopolygalacturonase	ELA28364.1	4	9	2.29	Degradation of pectin
Unknown						
D2	Not identified	–	–	–	∞	–
D3	Not identified	–	–	–	14.69	–
D4	Not identified	–	–	–	∞	–
D5	Not identified	–	–	–	∞	–

## Supplementary Materials

Supplementary materials can be found at http://www.mdpi.com/1422-0067/19/7/1860/s1.
