# Supplementary material for: BAS2 Is Required for Conidiation and Pathogenicity of Colletotrichum gloeosporioides from Hevea brasiliensis
Source: Int J Mol Sci. 2018 Jun 25;19(7):1860. doi: 10.3390/ijms19071860 (PMC6073657; doi:10.3390/ijms19071860)
Supplement: Supplementary file 1 [file ijms-19-01860-s001.pdf]

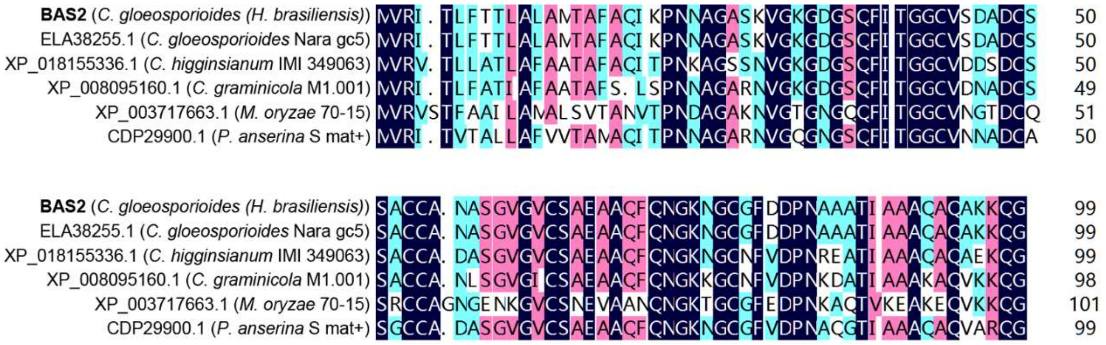

Figure S1. Alignment of amino acid sequences of BAS2 of *C. gloeosporioides* from *H. brasiliensis*, *C. gloeosporioides* Nara gc5, *C. higginsianum*, *C. graminicola*, *M. oryzae*, and *P. anserina*.

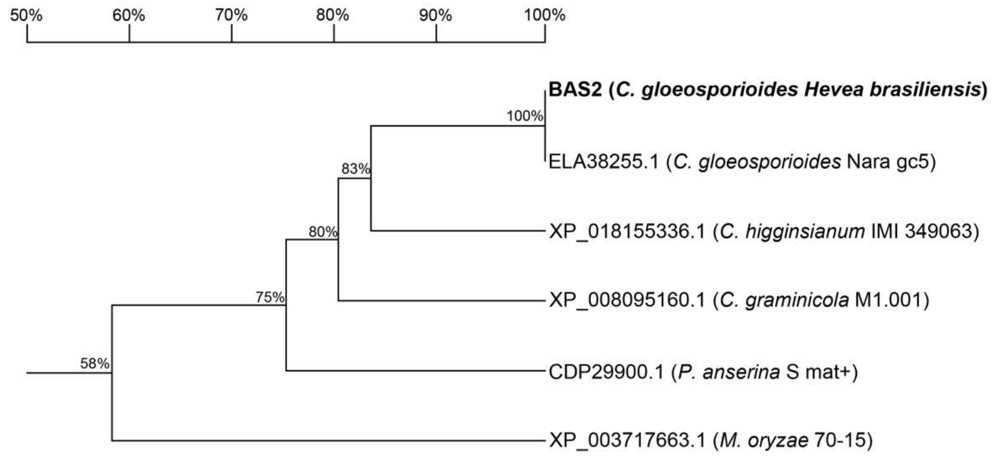

Figure S2. Phylogenetic analysis of BAS2 proteins. The neighbor joining phylogenetic tree was constructed by MEGA 7.0 according to the evolutionary relationship between BAS2 proteins in *C. gloeosporioides* from *H. brasiliensis*, *C. gloeosporioides* Nara gc5, *C. higginsianum*, *C. graminicola, M. oryzae*, and *P. anserina*.

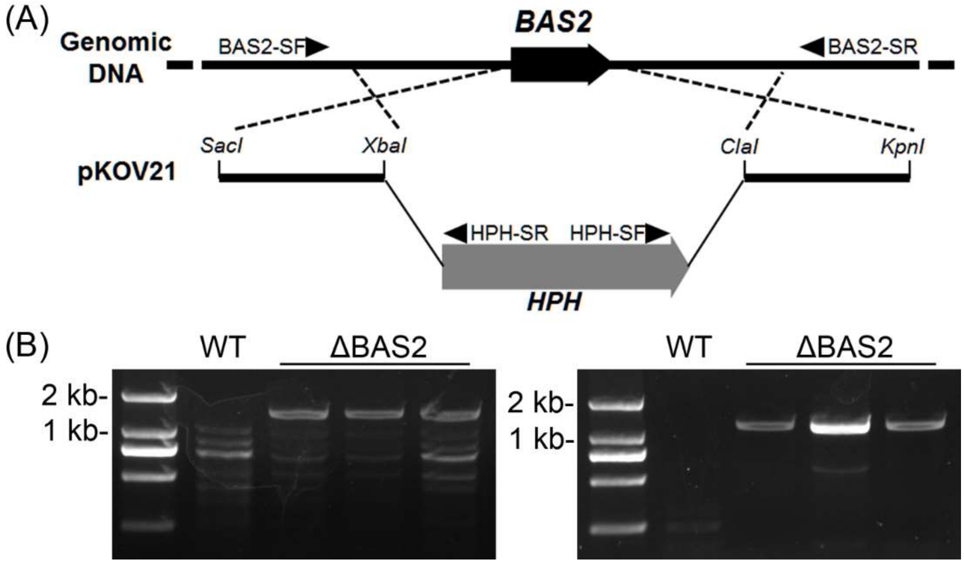

Figure S3. (A) The gene deletion strategy. Putative mutants were screened with diagnostic primers, indicated by black triangles. **(B)** Confirmation of the correct recombination of deletion cassettes with gene loci by Southern blot.

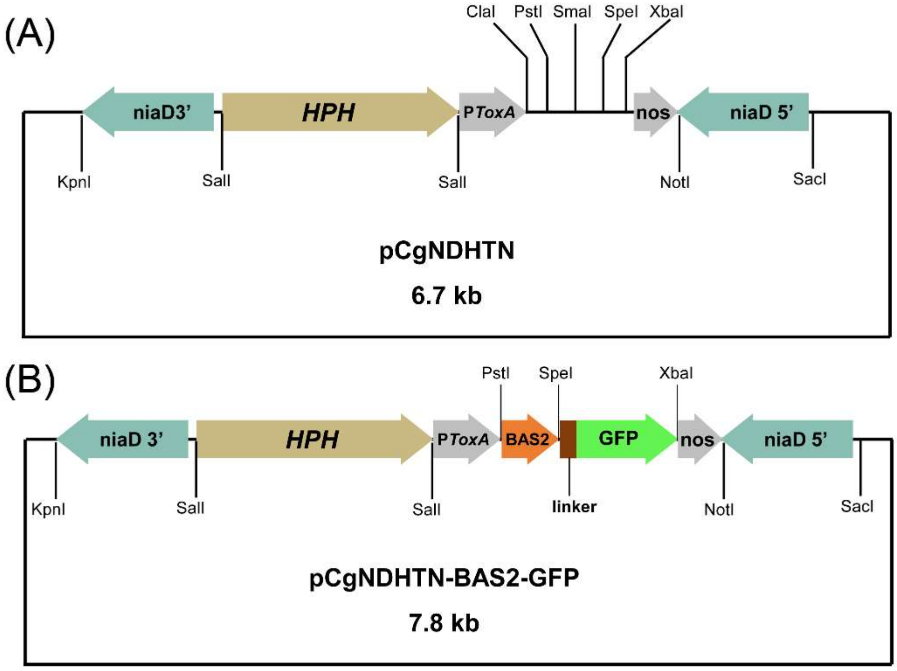

Figure S4. Strategy for generation of BAS2-GFP fusion overexpressing mutants. (**A**) Expression system using the gene loci of nitrate reductase (*niaD*). Promoter of ToxA and terminator nos were used for the gene expression; the Hygromycin phosphotransferase gene (*HPH*) was used for transformant selection. (**B**) The open reading frame of *BAS2* and the coding sequence of GFP with an N-terminal linker were linked together to construct the fusion expressing vector.

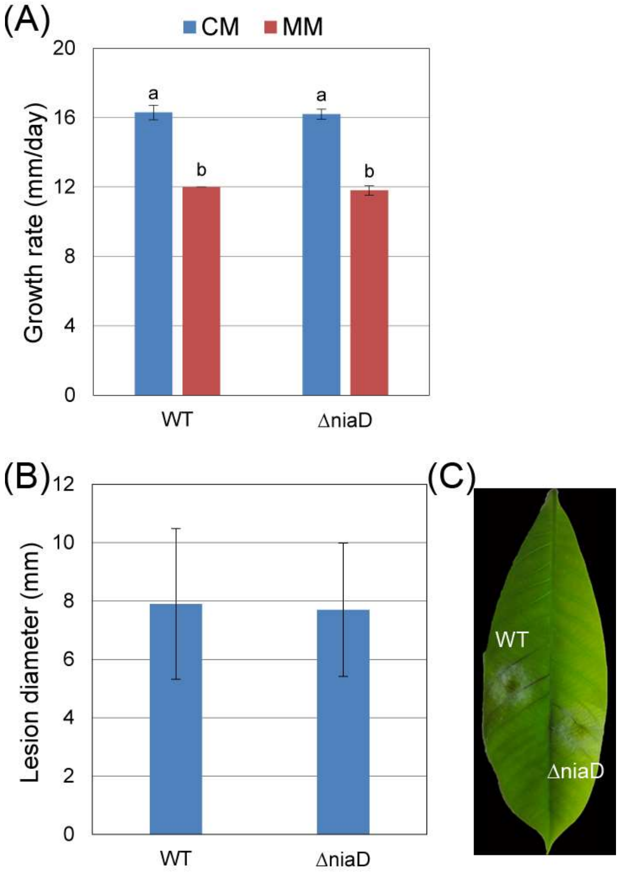

Figure S5. Phenotype assays of ΔniaD. **(A)** Growth rate assay of WT and ΔniaD cultured on complete medium (CM) and minimal medium (MM). **(B)** Mean lesion diameters after inoculation with conidia for 3 days. Bars represent standard deviation (SD). **(C)** Disease symptoms of rubber-tree leaves after inoculation with conidia suspension for 3 days.

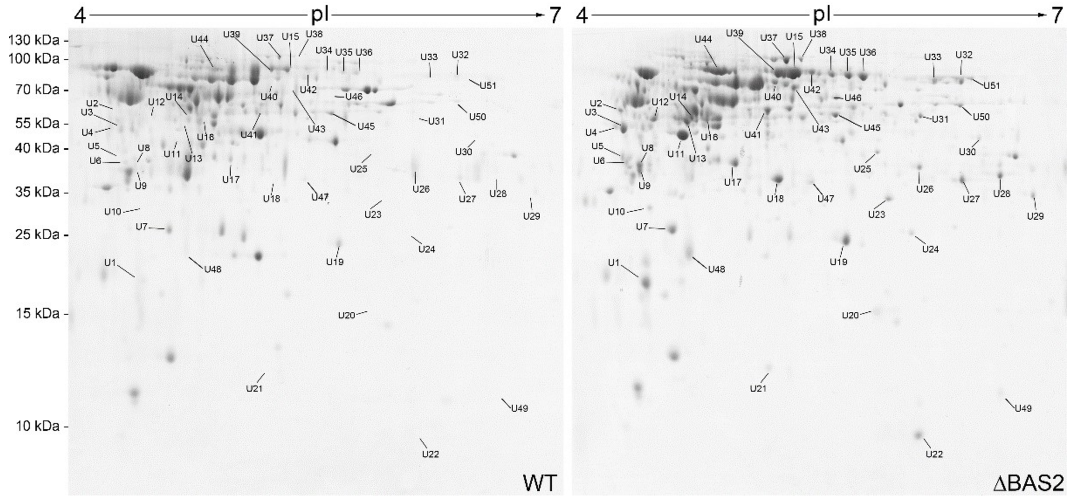

Figure S6. Two-dimensional patterns of extracellular proteomes of WT and ΔBAS2. Arrows indicate protein spots that were upregulated in abundance more than 1.5-fold between WT and ΔBAS2.

Table S1. PCR primers used in this study. Lowercase letters indicate induced restriction sites.

Number	Primer	Sequence (5′→3′)	Application
1	BAS2-5F	cgagctcTCGCAAAAATGTTCCAG	*BAS2* deletion
2	BAS2-5R	gctctagaTTTCGCGGTAGTTGAGTG	*BAS2* deletion
3	BAS2-3F	ccatcgatGCTGGAAATGCAGAAACT	*BAS2* deletion
4	BAS2-3R	ggggtaccAACATGGGGCAGGAGAC	*BAS2* deletion
5	BAS2-JC5F	GCGCATTCTTTGAGGTTTCTTG	ΔBAS2 diagnosis
6	HYG-JCR	TGAGTTCAGGCTTTTTCATTTGG	ΔBAS2 diagnosis
7	HYG-JCF	ACAGCGGTCATTGACTGGAGCGA	ΔBAS2 diagnosis
8	BAS2-JC3R	AAGGGCGGCGACAGTGAAGAGG	ΔBAS2 diagnosis
9	niaD-5F	gagctcAAGGAGTCCCGTTTGTT	*niaD* deletion, expression system
10	niaD-5R	gcggccgcACTGACGACTGGCTTGTC	*niaD* deletion, expression system
11	niaD-3F	gtcgacACGAGCTGCCGTTTTTAG	*niaD* deletion, expression system
12	niaD-3R	ggtaccCGGTCACGACGCTGTAA	*niaD* deletion, expression system
13	niaD-JC5F	TGCCAGTAGCGTGGTTTAGGTC	ΔniaD diagnosis
14	niaD-JC5R	tctagaAATTTCCCCGATCGTTC	ΔniaD diagnosis
15	niaD-JC3F	ACAGCGGTCATTGACTGGAGCGA	ΔniaD diagnosis
16	niaD-JC3R	AGTGTCCCAGATGTCGTGTTGC	ΔniaD diagnosis
17	Ptoxa-F	gtcgacTGGAATGCATGGAGGAG	Expression system of *C. gloeosporioides*
18	Ptoxa-R	atcgatGACCTATATTCATTCAT	Expression system of *C. gloeosporioides*
19	Tnos-F	tctagaAATTTCCCCGATCGTTC	Expression system of *C. gloeosporioides*
20	Tnos-R	gcggccgcCCGATCTAGTAACATAG	Expression system of *C. gloeosporioides*
21	HPH-F	gtcgacAACTGATATTGAAGGAG	Expression system of *C. gloeosporioides*
22	HPH-R	gtcgacAACTGGTTCCCGGTCGG	Expression system of *C. gloeosporioides*
23	cBAS2-F1	ctgcagATGGTCCGCATCACTCT	BAS2-GFP fusion expressing mutant
24	cBAS2-R1	actagtGAAACCTTGCTTCTTGG	BAS2-GFP fusion expressing mutant
25	GFPlink-F	actagtGGAGCTGGTGCAGGCGCTGGAGC CGGTGCCATGGTGAGCAAGGGCGA	BAS2-GFP fusion expressing mutant

26	GFP-R	tctagaTTACTTGTACAGCTCGT	BAS2-GFP fusion expressing mutant
27	cBAS2-F2	tctagaATGGTCCGCATCACTCT	Transient expression in rubber-tree protoplasts
28	cBAS2-R2	gagctctGAAACCTTGCTTCTTGG	Transient expression in rubber-tree protoplasts
